# Bleeding Ectopic Varices as the First Manifestation of Portal Hypertension

**DOI:** 10.1155/2014/140959

**Published:** 2014-09-22

**Authors:** Brij Sharma, Sujeet Raina, Rajesh Sharma

**Affiliations:** ^1^Department of Gastroenterology, Indira Gandhi Medical College, Shimla, Himachal Pradesh 171001, India; ^2^Department of Medicine, Dr. Rajendra Prasad Government Medical College, Kangra, Tanda, Himachal Pradesh 176001, India

## Abstract

Ectopic varices are defined as dilated portosystemic collateral veins in locations other than the gastroesophageal region. We present a case of recurrent upper gastrointestinal bleeding as the first manifestation of portal hypertension. We diagnosed ectopic duodenal varices without gastroesophageal varices on upper GI endoscopy and extrahepatic portal venous obstruction (EHPVO) on CT angiography and managed this case.

## 1. Introduction

Ectopic varices are defined as dilated portosystemic collateral veins in locations other than the gastroesophageal region [1, 2]. Ectopic varices account for 1% to 5% of all variceal bleeds in patients with intrahepatic portal hypertension and 20% to 30% of those with extrahepatic portal hypertension [1]. Ectopic varices have been reported to occur at numerous sites, including 18% in the jejunum or ileum, 17% in the duodenum, 14% in the colon, 8% in the rectum, and 9% in the peritoneum [3]. Ectopic varices present a clinical challenge as the etiology is multiple, localization is variable, modality of detection is heterogeneous, presentation is diverse, and management guidelines are not clear. We present a case of recurrent upper gastrointestinal bleeding, detected ectopic duodenal varices without gastroesophageal varices on upper GI endoscopy, and confirmed extrahepatic portal venous obstruction (EHPVO) on CT angiography. The case is presented to highlight the clinically challenging condition of ectopic varices, to have a high clinical suspicion to make the diagnosis especially in a patient with recurrent upper GI bleeding without history of chronic liver disease or stigmata of portal hypertension and normal upper GI endoscopy and may be the first manifestation of underlying portal hypertension as in our case.

## 2. Case History

**Figure 1:**
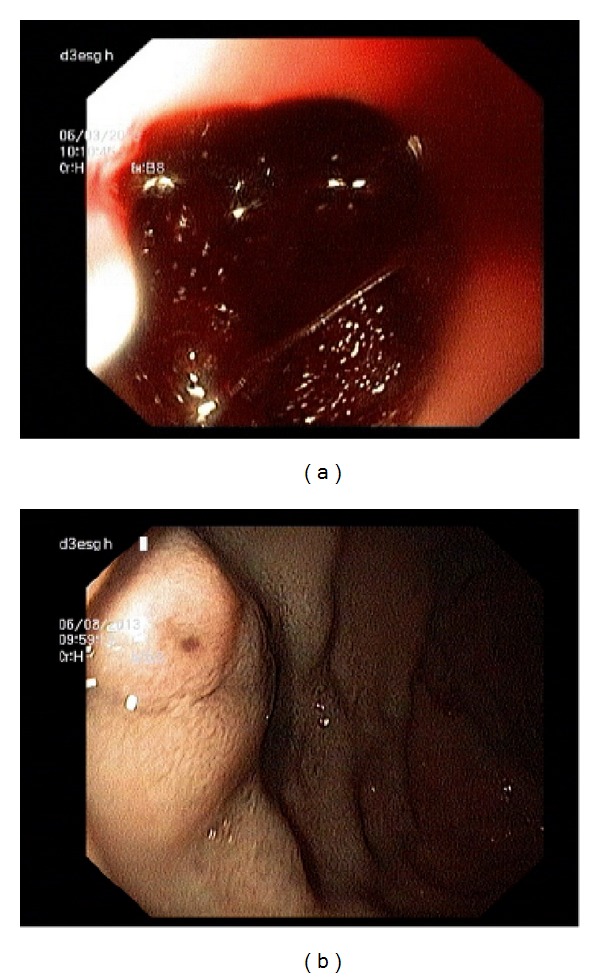
(a) Upper GI endoscopic image showing fresh blood in stomach. (b) Upper GI endoscopic image showing large duodenal varices.

**Figure 2:**
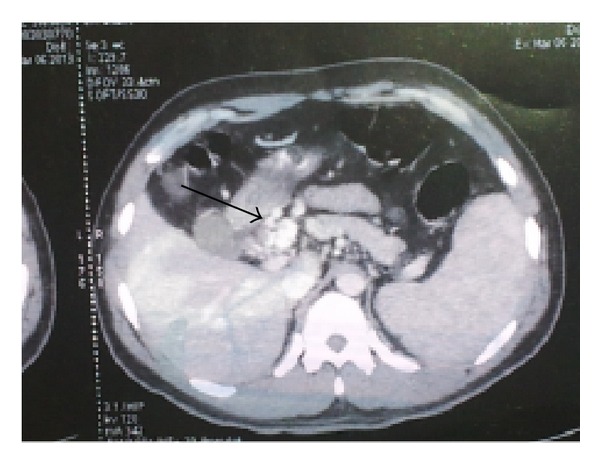
CT angiogram showing a coronal view of multiple collaterals (arrow) with poorly localized portal vein.

A 32-year-old male presented with history of hematemesis followed by melena for 3 days. History of dizziness on getting up from sitting position was present for one day. No history of pain abdomen, jaundice, drug intake, weight loss, or loss of appetite was present. In past history patient had two episodes of GI bleed in the last 2 years. Upper GI endoscopy outside this hospital was reported to be normal. There was no history of abuse or addiction. Review of other systems was normal. General physical examination revealed pallor and tachycardia and blood pressure measurement documented a postural fall of 20 mmHg. The rest of the general physical examination was normal and no stigmata of chronic liver disease were present. On systemic examination, spleen was palpable 2 cm below the left costal margin. The rest of the systemic examination was normal. On investigation, the hemoglobin was 7.6 gm% and platelet count was 1.6 lac/cmm. Blood glucose and renal functions were normal. Total serum bilirubin was 0.6 mg%. Serum total proteins were 6.3 gm% and albumin was 3.9 gm%. AST was 54 IU and ALT was 62 IU and alkaline phosphatase was 62 KAU. Upper GI endoscopy revealed fresh blood in the stomach (Figure 1(a)). A large varix with active spurt was observed in D1 part of duodenum (Figure 1(b)). One mL of N-butylcyanoacrylate was injected and homeostasis achieved. CT angiography demonstrated portal vein obstruction with multiple anomalous venous channels in region of portahepatis, periampullary region, and duodenum with mild splenomegaly (Figure 2). Thrombophilia (protein C, protein S, and antithrombin III) and antiphospholipid antibody work up was negative. Hyperhomocysteinemia was observed. Based on the clinical features and investigations the patient was diagnosed to have ectopic duodenal varices with portal hypertension with EHPVO. Patient was discharged on beta blocker and folic acid. Patient has undergone lienorenal shunt with splenectomy during followup.

## 3. Discussion

Esophagogastric varices are the most common complication in patients with portal hypertension while ectopic varices are less common [4]. Ectopic varices develop secondary to portal hypertension (PHT), surgical procedures involving abdominal organs and vessels, anomalies in venous outflow, abdominal vascular thrombosis, and hepatocellular carcinoma and may be familial in origin [5].

Ectopic varices account for up to 5% of variceal bleeds and present with hypovolemic shock, hematemesis, melaena, or hematochezia depending on the site of the varix [1, 5]. Ectopic varices should be strongly considered in patients with known liver disease or with stigmata of portal hypertension and upper gastrointestinal bleeding or hematochezia particularly when both upper and lower endoscopies fail to identify a source of bleeding [2]. Occult bleeding from ectopic varices may present with iron deficiency anaemia [1, 5].

Cirrhosis is the most commonly associated cause of duodenal varices, accounting for 30% of the cases. Duodenal varices have also developed after band ligation of esophageal varices and portal venous thrombosis and obstruction of the splenic vein and inferior vena cava. The most common afferent vessel for duodenal varices is the inferior pancreaticoduodenal vein (41%), followed by the superior mesenteric vein (10.2%), the duodenal vein (7.7%), and the superior pancreaticoduodenal vein (7.7%) [6]. Duodenal variceal rupture can lead to severe hemorrhage, with mortality as high as 40% from initial bleeding [7].

Management of ectopic varices includes initiation of resuscitation in the form of replacing intravascular volume loss by crystalloids and blood transfusion. Prophylactic antibiotics are recommended and vasopressors like terlipressin may be of benefit. Once patient is stabilized hemodynamically evaluate with emergency upper GI endoscopy. If it fails to show the source of bleeding, colonoscopy after a rapid colonic purge should be the next investigation [1, 5]. Intravenous contrast enhanced computed tomography (CT) may be preferable to colonoscopy (in the unprepared acute setting) and should be considered the primary modality for diagnosis of ectopic varices, given the rapid and ubiquitous availability [2]. Other modalities of investigation like color Doppler ultrasound have shown their value in the diagnosis of umbilical, duodenal, rectal, gall bladder, and choledochal varices.

In documented varices seen actively bleeding or having signs of recent bleed, endoscopic management with band ligation or endoscopic sclerotherapy or glue injection should be done. Secondary prophylaxis with ß-blockers is logical [5]. Interventional radiology treatment options after failed endoscopic techniques for duodenal varices include transjugular intrahepatic portosystemic shunt (TIPS), balloon occluded retrograde transvenous obliteration (B-RTO), and percutaneous transhepatic obliteration (PTO) [1, 5]. Surgery is a recommended option if endoscopic techniques and interventional radiologic procedures fail to control bleeding or are not feasible, provided the underlying good liver function and the local expertise. Surgery is preferred in patients with Child-Pugh “A” cirrhosis and in patients with an EHPVO [1, 5].

For secondary prophylaxis of bleeding in extrahepatic portal venous obstruction performing a lienorenal shunt with splenectomy carries a low operative mortality of 1%, a rebleeding rate of about 10%, shunt thrombosis rate of <2% over a 15-year followup, and survival rates of >96% after 10 years, is not followed by portosystemic encephalopathy and improves quality of life. Most importantly, it is a onetime procedure particularly suited to those who have little access to blood transfusion and sophisticated medical facilities [8].

To conclude, physicians need a high clinical index of suspicion to make the diagnosis of ectopic varices and be especially vigilant in patients with liver disease, as bleeding ectopic varices may be the first manifestation of underlying portal hypertension.
